# Are the Physical and Cognitive Functions of Older Adults Affected by Having a Driver’s License?—A Pilot Study of Suburban Dwellers

**DOI:** 10.3390/ijerph19084573

**Published:** 2022-04-11

**Authors:** Keisuke Itotani, Ippei Suganuma, Hiroyuki Fujita

**Affiliations:** 1 Department of General Rehabilitation, Faculty of Allied Health Sciences, Yamato University, 2-5-1 Katayama-cho, Suita 564-0082, Osaka, Japan; 2 Department of Occupational Therapy, Faculty of Health Sciences, Kyoto Tachibana University, 34 Oyakeyamada-cho, Yamashina-ku, Kyoto 607-8175, Japan; suganuma-i@tachibana-u.ac.jp; 3 Department of Physical Therapy, Faculty of Health Care, Osaka University of Human Sciences, 1-4-1 Shoujaku, Settsu 566-8510, Osaka, Japan; h-fujita@kun.ohs.ac.jp

**Keywords:** community-dwelling older adults, non-driver’s license, cognitive function

## Abstract

Previous studies have frequently reported that those with a driver’s license have better physical and cognitive functions than those without. However, there are many people in the world who do not need or who cannot have a driver’s license. We hypothesized that if the non-driver’s license group had the same or better physical and cognitive functioning as the driver’s license group, they could lead healthy lives without the risk of functional decline or loss of functioning due to surrendering their licenses or giving up driving. The subjects were 47 community-dwelling older adults. We measured their physical function and cognitive function and performed psychological assessment via the following tests: grip strength, Timed Up and Go test, walking speed, Five Times Sit to Stand test, Functional Reach test, Two-Step Test, Mini-Mental State Examination, Trail Making Test, Modified Falls Efficacy Scale, Geriatric Depression Scale, and University of California Los Angeles Loneliness Scale. In previous studies, it has been said that having a driver’s license provides good physical, cognitive, and psychological functions. However, in this study, loneliness and executive function were strongly influenced by age and sex, and no direct relationship to a driver’s license was suggested. Rather, non-driver license holders may be relieved because there is no risk of accidents due to driving, and there is no possibility of a suddenly decline in physical or cognitive function due to revocation of a driver’s license.

## 1. Introduction

In 2018, 18.63 million (52.2%) of the 35.71 million older adults aged 65 and over in Japan had a driver’s license [1,2]. It has been clarified that, in older adults, possession of a driver’s license has the positive effects of increasing physical activity and having relatively good executive function [3,4]. In addition, it has been reported that car ownership and driving are highly correlated with the independence and life satisfaction of older adults [5,6,7,8]. In developed countries, driving is the preferred means of transportation for individuals and is controlled as part of their lives [9,10,11], making it an indispensable tool for their social role. In particular, Fricke et al. identified driving in older adults as the second most important activity in the IADL task [12].

Furthermore, it has been clarified that older adults experience a decrease in physical ability and cognitive function as they age, which makes it difficult to drive a car and increases the risk of causing an accident [13]. In Japan, annual data from 2010 to 2020 shows an increase in the percentage of all accidents involving older adult drivers, over the age of 65, in all age groups [14]. Decreased driving skills are significantly associated with impaired executive and memory functions, including generalized cognitive function [15,16]. In addition, in older adults, infrequent driving and returning an automatic driver’s license may adversely affect health [17,18] and physical activity [19]. It has been reported that these outcomes tend to be similar in Japan; they lead to a high risk of long-term care [20] and are a cause of cognitive decline [21]. On the other hand, 43.1% of older Japanese adults aged 65 and over do not have a driver’s license. The number of young Japanese people who do not have a driver’s license is increasing [1,2], so there is a possibility that the number of older adults who do not have a driver’s license will increase in the future. Globally, there are numerous reasons why people may have never obtained a driver’s license, for example, always living in a metropolitan area where public transportation is relatively convenient and vehicle ownership is expensive and troublesome, for cultural reasons such as some women being forbidden or discouraged to drive due to trends of the era or for religious reasons, or for economic and educational reasons, among others. Lack of a driver’s license does not mean that older adults have difficulty with independence in their daily lives or are in trouble. It is likely that they have used public transportation in their daily lives to shop and go out. A previous study [22] reported that older adults participate more frequently in social activities when they are unable to drive and when they have access to private or public transportation. It has also been reported that community transport using public transportation is favorable to older adults’ health [23]. Therefore, it is presumed that these skills will become habitual if they are continued for a long period of time and that physical function and cognitive function will be maintained relatively well. However, there are no reports focusing on such non-driver’s license holders, but there are many reports that consistently show the superiority of driver’s license holders [1,2,3,4,5,6,7,8,9,10,11,12].

If older adults who do not have a driver’s license have the same, or better, physical and cognitive functions as older adults who do have one, then there would be no deterioration or loss of physical function caused by the return of the license, which is peculiar to the older adults who possess one. In that case, it can be assumed that the older adults without a license can continue to live a healthy life. Therefore, the purpose of this study is to investigate the characteristics of the physical and cognitive functions of non-driver’s license holders (who have never held a driver’s license) among older adults living in the community, as a pilot study, and the characteristics of such non-driver’s license holders.

## 2. Materials and Methods

Physical and cognitive functioning was measured in older adults living in the city of Suita from August to December 2019. The recruitment and exclusion criteria for these measures were carried out in the same way as a previous study [24]. In brief, older community-dwelling adults were recruited approximately two months before the testing session by information leaflets and posters provided at community centers. Exclusion criteria for analysis were as follows: (1) less than 65 years old, (2) consent was not obtained, (3) all measurements were not performed completely, or (4) analysis data was missing. Analysis was performed on the data of 57 people aged from 61 to 90 who met the criteria. Information about this study was provided in writing to all the subjects prior to starting the assessment, and all subjects provided their informed consent. This study was approved by the Research Ethics Committee of the Faculty of Health Sciences, Yamato University.

### 2.1. Demographic Data

Demographic data including age, gender, height, weight, educational history (number of years from elementary school to last institution of education), number of people living with the subject, alcohol consumption status (do not drink, rarely, sometimes, every day), history of falling, daily activity time (time to exclude: sleep time, sitting time, lying time), hobbies, possession of a driver’s license, and comorbidities were obtained from a self-administered questionnaire. A history of falling in the past year was defined as “unintentionally coming to the ground or some lower level and other than as a consequence of sustaining a violent blow, loss of consciousness, sudden onset of paralysis as in stroke or an epileptic seizure during the past 1 year” [25]. Comorbidities were evaluated by self-reported answers written by the subjects on their assessment sheet and included hypertension, diabetes, hip osteoarthritis, knee osteoarthritis, and spinal canal stenosis. At the time of assessment, those who currently possessed a driver’s license were defined as the driver’s license group (D group), while those who did not currently possess a driver’s license and had never possessed a driver’s license in the past were defined as the non-driver’s license group (ND group).

### 2.2. Physical Function and Performance Measurements

We performed five physical tests: grip strength, the Timed Up and Go test (TUG), the Short Physical Performance Battery (SPPB), a Functional Reach Test (FRT) and a Two-Step Test. Grip strength was measured by a Smedley-Type Hand Dynamometer Grip D (Takei Kiki Kogyo, Tokyo, Japan). Measurements were carried out twice on the dominant hand, and a maximum amount of 2 trials was used for analysis. The exact methods of TUG [26], SPPB [27], and FRT [28] were carried out in the same way as our previous studies [24,29] with reference to other studies [26,27,28]. The score of the SPPB was calculated according to the method of Guralnik et al. [27] (12 points perfect score). The Two-Step Test is one of the locomotive syndrome risk tests advocated by the Japanese Orthopaedic Association [30]. First, the subject stands with the toes on both sides at the start line. They then walk two steps, as lengthy as possible, and stop with both feet together. The length of the two steps from the start line to the tip of the toes in the end position is measured. The Two-Step Test score was calculated as the two-step distance (cm)/height (cm) [31]. Each physical test was performed twice, and the average value was calculated for each.

### 2.3. Cognitive Function Measurements

Cognitive functions were measured using a Mini-Mental State Examination (MMSE), and the Trail Making Test Part A (TMT-A) and Part B (TMT-B). The MMSE consists of a 0 to 30 score by short-term memory registration and recall, attention, naming, following verbal commands, judgment, and copying a double pentagon. Subjects with an MMSE score of 20–24 are defined as having mild cognitive impairment [32]. The TMT was conducted in accordance with Wagner et al. [33], and the procedure for the tests and analysis follows that of our previous study [24]. In addition, the time difference between TMT-A and TMT-B (ΔTMT) was also used.

### 2.4. Psychological Assessment

The Modified Falls Efficacy Scale (M-FES) [34] consists of Activities of Daily Living (ADL) and Instrumental Activities of Daily Living (IADL). This assessment can be ex-pressed as a score of fear of falling for indoor and outdoor activities. In this study, subjects were asked to answer in four stages (1 = I am not confident at all, 2 = I am not very confident, 3 = I am a little confident, 4 = I am very confident) so that the subjects could easily understand. The score is a 14–56 score, and the higher the score, the stronger the self-confidence in ADL. The Geriatric Depression Scale (GDS) [35] answers 15 questions with “yes” or “no”, and the score is 0–15. This is a question index indicating that the higher the score, the stronger the tendency toward depression. The classification is defined as a 0–4 score as normal (negative), and a score of 5 or more as depressed (positive) [36]. The University of California Los Angeles Loneliness Scale Version 3 (UCLA) [37] consists of 20 items that assess social isolation and loneliness. Each item is evaluated on a scale from 1 (not at all) to 4 (frequently). The higher the score, the higher the feeling of loneliness.

### 2.5. Hobbies Assessment

In the hobbies assessment, we referred to Matsutsuyu’s classification [38]. It is a hob-by activity divided into eight categories (sports activity, cultural activity, musical activity, creative activity, horticultural activity, audiovisual activity, tourism activity, investment, and gambling activity). The presence or absence was investigated, and the score (0–8 points) was calculated with “Yes = 1 point” and “No = 0 point”.

### 2.6. Statistical Analysis

Subjects’ assessment variables were analyzed using the student’s *t*-test and Chi-square test. Furthermore, comparisons were made using an analysis of covariance (AN-COVA) adjusted for variables for which significant differences were observed in the demographic data. The significance level was set at *p* < 0.05. Analysis was performed using IBM SPSS version 26 (IBM Corporation, Armonk, NY, USA).

## 3. Results

The area where all subjects lived has three types of highway nodes, and train stations and airports are located within 10 km of the central city area. National roads, arterial roads and multiple railroad lines pass through the city, facilitating movement to and from the city center and neighboring cities. Various public facilities, such as community facilities, nursery centers, children’s halls, welfare facilities, social education facilities, and physical education facilities, are located in the area.

Of the 57 subjects, 45 (78.9%) were included in the analysis (12 had a driver’s license in the past but were not current drivers at the time of the study and were excluded) (Table 1). Of the 45 subjects, 18 (40%) were in the D group, and 27 (60%) were in the ND group. The results of the student’s t-test and Chi-square test showed that the ND group was of a significantly older age (*p* < 0.001, r = −0.843), included more females (*p* = 0.002, φ = −0.48), had a weaker grip strength (*p* = 0.002, r = 1.023), had a slower speed for the TUG speed (*p* = 0.03, r = −0.645), had a shorter distance for the FRT (*p* = 0.004, r =.925), had a slower speed for the TMT-B (*p* = 0.046, r = 0.30), had a higher time for ∆TMT (*p* = 0.049, r = 0.29), had a lower score for the M-FES (*p* = 0.009, r = 0.701) and had a higher score for the UCLA (*p* = 0.007, r = −0.862) than those of the D group (Table 2). The results of the demographic data showed a significant difference between the ND group and the D group in the variables of age and sex. Therefore, ANCOVA was conducted using these two variables as adjustment factors. ANCOVA results showed that the ND group had a significantly shorter daily activity time (*p* = 0.049, r = 0.099), slower time for 5CS (*p* = 0.042, r = 0.105), shorter distance for the FRT (*p* = 0.024, r = 0.123) and lower score for the M-FES (*p* = 0.046, r = 0.099) than the D group (Table 3).

## 4. Discussion

This study compared the physical and cognitive functioning of the ND group and D group among community-dwelling older adults. We hypothesized that if the ND group had the same or better physical and cognitive functioning as the D group, they could lead healthy lives without the risk of functional decline or loss of functioning due to surrendering their licenses or not driving. First, when we investigated the ratio of driver’s license holders by age in Japan [1], we found that there are many people over 80 years old in terms of the age ratio of driver’s license holders. Secondly, the results showed that the ND group was characterized by being of an older age, having a higher proportion of females, a weaker grip strength, poor standing balance and executive function, and a higher fear of falling and loneliness. Furthermore, the results of ANCOVA showed that the ND group had a shorter daily activity time, lower limb muscle strength and balance, and a higher fear of falling than the D group. However, there were no significant differences in grip strength, executive function, or loneliness, which were significant before adjustment.

In previous studies, it was reported that older adults with a driver’s license had relatively good physical activity and executive function [3,4]. In this study, as a result of adjusting by age and sex, it was clarified that the D group had a significantly longer daily activity time, stronger lower limb muscle strength, and better balance ability than the ND group. This result partially supports previous studies. However, executive function did not differ significantly between the ND group and the D group, indicating that it was not related to the possession of a driver’s license. Decline in executive function has been shown to be associated with old age [24], and, in this study, it was also strongly influenced by age. In addition to age, gender was also found to have an effect. Therefore, maintaining executive function by possessing a driver’s license, or executive function declining due to non-possession, is considered unlikely to occur. Alternatively, it has been reported that a decline in driving skills is mainly associated with a decline in executive function [15,16]. If an older adult driver has a decline in driving skills, it is advisable to recognize that executive function is impaired. In addition, for driver’s license holders, there will come a time in the future when driving licenses will be revoked or surrendered for one reason or another, or when driving will no longer be an everyday activity. These occurrences can lead to a rapid decline in physical and cognitive function, so driver’s license holders should take care. 

Secondly, regarding the relationship between driving, activity, and mental health, in developed countries, driving is a part of life [9,10,11] and is an important activity in the IADL task of older adults [12]. In addition, car ownership and driving are highly correlated with the independence and life satisfaction of older adults [5,6,7,8]. The impact of a driver’s license is not only on people’s lives, but also on their mental health. In this study, the M-FES score in the ND group was significantly lower than that in the D group. This indicates that the ND group is more afraid of falls. These results supported previous research that possession of a license has a positive effect on mental health. Furthermore, according to previous studies, it was reported that about 19% acknowledged avoiding activities because of fear of falling [39], and, of those with a fear of falling, 56% had curtailed activity due to this fear [40]. With these in mind, the reason why the daily activity of the ND group in this study was less may be due to the fear of falling.

Regarding loneliness, previous studies have reported that the ability to spend time alone may not be negatively affected [41]. Since the subject of this study could come to the measurement venue by themselves, it seems that their ADL is independent. However, since the level of independence in daily life decreases with aging, it is considered that the feeling of loneliness may be influenced not by the possession of a driver’s license but by age. 

This study investigated the effects of the possession of a driver’s license on the physical, cognitive, and psychological functions of community-dwelling older adults compared to non-driver’s license holders. However, this study has some limitations. Because subjects in this study were voluntary subjects who came to the measurement venue, results may differ for other older subjects, such as patients in hospitals and institutions. Second, the demographic data was written by the subject themselves and may not have been accurate. Third, the subjects of this study lived in areas that are densely populated and which have sufficient public transportation such as buses and trains. Further study must make a comparison that also includes the environment of the area of residence, including areas in which driving is necessary for daily needs such as grocery shopping, etc. In the future, in addition to expanding the study area and increasing the number of subjects, it will be necessary to conduct longitudinal studies of subjects in other places, such as hospitals and institutions.

## 5. Conclusions

In this study, we did not find any factors that made the ND group superior to the D group, but it was found that loneliness and executive function were strongly influenced by age and sex. Therefore, non-drivers do not feel reluctant about not having a driver’s li-cense. Rather, there is no risk of accidents due to driving, and there is no possibility that physical function and cognitive function will suddenly decline due to revocation or return of the license; therefore, it may be valuable to think positively about this. This study provides an opportunity to explore the advantages and disadvantages of possession of a driver’s license and may help to address the issue of license surrender among older adults living in the community.

## Figures and Tables

**Table 1:** Characteristics of subjects.

	Mean (SD) or Number (%)							
	Total		D Group		ND Group		*p* Value	Effect Size
Variables	*n* = 45		*n* = 18		*n* = 27			(*r*, *φ*)
Age, y	76.7	(6.6)	73	(8.3)	78.6	(5.2)	0.001 ^a^	−0.843
Sex, male	13	(28.9)	10	(55.6)	3	(11.1)	0.002 ^b^	−0.480
Height, cm	155.5	(8.8)	162	(9.8)	152.4	(6.4)	0.001 ^a^	1.213
Weight, kg	55.5	(9.9)	62	(11.6)	53.3	(7.8)	0.004 ^a^	0.915
BMI, kg/m^2^	22.9	(2.8)	23.6	(3.5)	22.8	(2.2)	n.s.	.0266

Abbreviations: BMI, body mass index; n.s., not significant. ^a^, Student’s *t*-test; ^b^, Chi-square test.

**Table 2:** Comparison between the two groups: D group and ND group.

Crude Model	Mean (SD) or Number (%)							
	Total		D Group		ND Group		*p* Value	Effect Size
Variables	*n* = 45		*n* = 18		*n* = 27			(*r*, *φ*)
Education, y	12.3	(2.6)	12.8	(3.5)	11.6	(1.8)	n.s.	0.432
No. of people living together, n	2.2	(1.3)	2.2	(1.3)	2.2	(1.5)	n.s.	−0.013
Living alone, yes	13	(28.9)	5	(27.8)	8	(29.6)	n.s.	−0.020
Alcohol consumption status							n.s.	0.236
not drink	24	(53.3)	8	(44.4)	16	(59.3)		
rarely	7	(15.6)	3	(16.7)	4	(14.8)		
sometimes	4	(8.9)	1	(5.6)	3	(11.1)		
every day	10	(22.2)	6	(33.3)	4	(14.8)		
Activity time, h/day	7.6	(3.2)	8.2	(3.8)	7.3	(2.5)	n.s.	0.13
History of falling, yes	11	(24.4)	5	(27.8)	6	(22.2)	n.s.	0.063
No. of hobby, n	4.6	(1.7)	5.1	(1.2)	4.3	(1.9)	n.s.	0.23
Hypertension, yes	21	(46.7)	8	(44.4)	13	(48.1)	n.s.	−0.036
Diabetes, yes	4	(8.9)	0	(0)	4	(14.8)	n.s.	−0.255
Hip OA, yes	2	(4.4)	1	(5.6)	1	(3.7)	n.s.	0.044
Knee OA, yes	8	(17.8)	3	(16.7)	5	(18.5)	n.s.	−0.024
Spinal canal stenosis, yes	4	(8.9)	3	(16.7)	1	(3.7)	n.s.	0.223
VCF, yes	0	(0)	0	(0)	0	(0)	n.s.	-
Osteoporosis, yes	9	(20.0)	2	(11.1)	7	(25.9)	n.s.	−0.181
Grip strength, kg	24.4	(7.8)	29.5	(9.6)	21.9	(5.5)	0.002 ^a^	1.023
TUG, s	7.9	(1.9)	7	(1.5)	8.2	(2.0)	0.03 ^a^	−0.645
SPPB, score	11.6	(0.7)	11.7	(0.7)	11.6	(0.8)	n.s.	0.04
Walking speed, m/s	1.8	(0.4)	1.7	(0.5)	1.9	(0.4)	n.s.	−0.357
Tandem standing, possible	42	(93.3)	16	(88.9)	26	(96.3)	n.s.	−0.145
5CS, s	8.6	(2.8)	7.5	(2.0)	8.9	(2.8)	n.s.	−0.56
FRT, cm	24.2	(5.7)	27.6	(4.7)	22.3	(6.3)	0.004 ^a^	0.925
2-step test, score	1.31	(0.2)	1.35	(0.3)	1.29	(0.2)	n.s.	0.279
MMSE, score	28.6	(2.0)	28.7	(2.0)	28.5	(2.0)	n.s.	0.073
TMT-A, s	86.4	(33.3)	78.3	(19.5)	87.4	(30.6)	n.s.	0.19
TMT-B, s	143.4	(73.7)	122.2	(56.2)	155	(76.2)	0.046 ^b^	0.30
⊿∆TMT, s	57.7	(53.7)	44	(49.1)	67.6	(60.1)	0.049 ^b^	0.29
M-FES, score	52	(6.0)	55.3	(1.4)	51.6	(6.7)	0.009 ^a^	0.701
GDS, score	3.4	(3.1)	2.4	(2.5)	3.3	(2.9)	n.s.	−0.318
UCLA, score	34.8	(9.3)	29.7	(6.8)	37.2	(9.7)	0.007 ^a^	−0.862

Abbreviations: D group, driver group; ND group, non-driver group; OA, osteoarthritis; VCF, vertebral compression fracture; TUG, Timed Up and Go test; SPPB, Short Physical Performance Battery; 5CS, 5 Chair Stand; FRT, Functional Reach Test; MMSE, Mini-Mental State Examination; TMT-A, Trail Making Test Part A; TMT-B, Trail Making Test Part B; M-FES, Modified Falls Efficacy Scale; GDS, Geriatric Depression Scale; UCLA, University of California Los Angeles Loneliness Scale Version 3; n.s., not significant. ^a^, Student’s *t*-test; ^b^, Mann–Whitney U test.

**Table 3:** Comparison between the two groups adjusted for age and sex.

Adjusted Model	Mean (SD) or Number (%)							
	Total		D Group		ND Group		*p* Value	Effect Size
Variables	*n* = 45		*n* = 18		*n* = 27			(*η^2^*)
Education, y	12.1	(2.7)	12.8	(3.6)	11.6	(1.8)	n.s.	0.004
No. of people living together, n	2.2	(1.4)	2.2	(1.3)	2.2	(1.5)	n.s.	0.054
Activity time, h/day	7.5	(3.0)	7.9	(3.6)	7.3	(3.0)	0.049	0.099
No. of hobby, n	4.6	(1.7)	5.0	(1.2)	4.3	(1.9)	n.s.	0.007
Grip strength, kg	25.1	(8.4)	29.9	(9.8)	22.0	(5.6)	n.s.	0.008
TUG, s	7.8	(1.9)	7.1	(1.5)	8.3	(2.0)	n.s.	0.056
SPPB, score	11.6	(0.7)	11.5	(0.8)	11.7	(0.7)	n.s.	0.004
Walking speed, m/s	1.9	(0.5)	1.8	(0.4)	1.9	(0.4)	n.s.	0.001
5CS, s	8.4	(2.6)	7.5	(2.0)	9.0	(2.7)	0.042	0.105
FRT, cm	24.3	(6.4)	27.7	(4.9)	22.2	(6.4)	0.024	0.123
2-step test, score	1.3	(0.2)	1.3	(0.3)	1.3	(0.2)	n.s.	0.052
MMSE, score	28.5	(2.0)	28.6	(2.1)	28.5	(2.1)	n.s.	0.026
TMT-A, s	85.3	(26.4)	80.0	(19.3)	89.1	(30.0)	n.s.	0.034
TMT-B, s	145.3	(70.0)	125.1	(56.6)	158.5	(75.5)	n.s.	0.002
⊿∆TMT, s	60.0	(57.3)	45.5	(50.2)	69.4	(60.6)	n.s.	0.001
M-FES, score	53.0	(5.6)	55.3	(1.4)	51.5	(6.8)	0.046	0.099
GDS, score	2.9	(2.7)	2.1	(2.3)	3.4	(2.9)	n.s.	0.021
UCLA, score	34.2	(9.3)	30	(6.9)	37	(9.8)	n.s.	0.037

Abbreviations: D group, driver group; ND group, non-driver group; TUG, Timed Up and Go test; SPPB, Short Physical Performance Battery; 5CS, 5 Chair Stand; FRT, Functional Reach Test; MMSE, Mini-Mental State Examination; TMT-A, Trail Making Test Part A; TMT-B, Trail Making Test Part B; M-FES, Modified Falls Efficacy Scale; GDS, Geriatric Depression Scale; UCLA, University of California Los Angeles Loneliness Scale Version 3; n.s., not significant. Comparisons were made using analysis of covariance (ANCOVA).

## Data Availability

Not applicable.
